# Dynamic Changes in Host Gene Expression following *In Vitro* Viral Mimic Stimulation in Crocodile Cells

**DOI:** 10.3389/fimmu.2017.01634

**Published:** 2017-11-22

**Authors:** Subir Sarker, Yinan Wang, Brenden Warren-Smith, Karla J. Helbig

**Affiliations:** ^1^Department of Physiology, Anatomy and Microbiology, School of Life Sciences, La Trobe University, Melbourne, VIC, Australia; ^2^Genomics Research Platform, La Trobe University, Melbourne, VIC, Australia

**Keywords:** crocodile, RNA-sequencing, transcriptome analysis, virus, immune response, innate immunity, interferon, reptile

## Abstract

The initial control of viral infection in a host is dominated by a very well orchestrated early innate immune system; however, very little is known about the ability of a host to control viral infection outside of mammals. The reptiles offer an evolutionary bridge between the fish and mammals, with the crocodile having evolved from the archosauria clade that included the dinosaurs, and being the largest living reptile species. Using an RNA-seq approach, we have defined the dynamic changes of a passaged primary crocodile cell line to stimulation with both RNA and DNA viral mimics. Cells displayed a marked upregulation of many genes known to be involved in the mammalian response to viral infection, including viperin, Mx1, IRF7, IRF1, and RIG-I with approximately 10% of the genes being uncharacterized transcripts. Both pathway and genome analysis suggested that the crocodile may utilize the main known mammalian TLR and cytosolic antiviral RNA signaling pathways, with the pathways being responsible for sensing DNA viruses less clear. Viral mimic stimulation upregulated the type I interferon, IFN-Omega, with many known antiviral interferon-stimulated genes also being upregulated. This work demonstrates for the first time that reptiles show functional regulation of many known and unknown antiviral pathways and effector genes. An enhanced knowledge of these ancient antiviral pathways will not only add to our understanding of the host antiviral innate response in non-mammalian species, but is critical to fully comprehend the complexity of the mammalian innate immune response to viral infection.

## Introduction

The innate immune system carries a substantial burden of defense against viral pathogens. The study of this response across animal species in recent years, as well as the examination of the phylogenetic conservation of these responses has changed our concept of innate immunity; however, much of this work has been performed in mammals. Major explorations of antiviral innate immune responses in non-mammalian species remains very limited, but may be critical to fully comprehend the complexity of the mammalian innate immune response to viral infection, and the discovery of novel antiviral therapeutics (1).

Mammalian cells have been shown to orchestrate elaborate defense mechanisms to detect and inhibit viral replication. Immediately after viral sensing by the host cells, the innate immune response is initiated by germline-encoded molecules termed pattern recognition receptors (PRRs). PRPs recognize conserved features of viruses and other microorganisms, known as pathogen-associated molecular patterns (PAMPs), which are small molecular motifs recognized as non-self, such as microbial nucleic acids, proteins, and carbohydrates (2). Following the recognitions of PAMPs, PRRs initiate a set of signaling cascades, which ultimately result in the production of interferon (IFN) and the upregulation of hundreds of interferon-stimulated genes (ISGs) (3). The expression of these ISGs is known to limit pathogens, in particularly viral pathogens, although the exact role of the majority of these ISGs remains unknown (4), more specifically we have very little understanding of how this response is orchestrated in non-mammalian vertebrate species.

There is an enormous lack of information surrounding antiviral innate immunity in the *Reptilia* class, which represents a bridge between fish and mammals. Both type I and type III IFNs are known to be central cytokines in the antiviral response in mammals, inducing the upregulation of hundreds of antiviral effector genes (5). To date, both functional type I and III IFNs have been found in the genomes of amphibians (6, 7), with type I IFNs also being found in fish (8). In reptiles, type III IFN has recently been found in the genome of lizards (9); however, the pathways that upregulate their production have not been described to date, and there is very little information on the downstream antiviral effector genes that may be responsible for viral control in reptiles. Recent transcriptomics work performed on non-infected tissue in the lizard has been able to identify the presence of multiple known PRRs in the reptile (10) with recent studies by our group also showing a number of known ISGs to be upregulated in the presence of viral infection in a reptile *in vitro* (11). The ISGs, viperin, 2′-5′-oligoadenylate synthetase (OASL) and IFN-induced GTP-binding protein Mx1 were demonstrated to be upregulated in saltwater crocodile, *C. porosus* LV-1 cells in the presence of the viral mimics, dsRNA, and dsDNA, as well as in the presence of replicating dengue virus (11). This study also demonstrated that crocodile viperin retained its antiviral activity and was able to inhibit dengue viral replication *in vitro*. Given the large number of viruses described to infect reptiles (12), these studies only give a very small insight into the induction of antiviral pathways in the reptile.

The world’s largest living reptile species, the saltwater crocodile, is a member of the prehistoric order *Crocodylia*, evolved from the archosauria clade that includes the dinosaurs, pterosaurs, crocodilians, and birds, the latter two being the only extant members of the clade (13). In recent years, two novel herpesviruses, crocodyline herpesvirus 1 and crocodyline herpesvirus 2 (CrHV-1 and -2, respectively) (14, 15), adenovirus, and poxvirus have emerged as significant viral infections of the crocodile, with multiple bacterial and fungal pathogens also being detected in this saltwater crocodile (16). However, very little is known about the ability to control these pathogens by the host immune system. The recent advancement of the crocodilian genome sequence (17) has given the opportunity to unveil the innate immune pathways in this ancient species, in particular its response to viral pathogens.

In recent years, transcriptomes profiling using high-throughput RNA sequencing (RNA-seq) technology has provided unprecedented opportunities to study the host response to infection against a wide range of viral and bacterial infections (18–22). RNA-seq technology is both an efficient and accurate tool to reveal the systemic changes in host gene expression in response to infectious pathogens, which could help to unearth a better understanding of innate immune pathways in *Reptilia*. In the present study, we have used RNA-seq technology to comprehensively study the host transcriptomic profile following viral mimic stimulation of crocodile cell lines using both dsRNA and dsDNA. This study provides a global view for the first time, of nucleic acid-specific and post-stimulation time-specific mRNA profiles in the saltwater crocodile (*C. porosus*), adding significantly to the body of knowledge surrounding the early innate host response of reptiles to a virus.

## Materials and Methods

### Cells Stock and Stimulation Using dsRNA and dsDNA

The *Crocodylus porosus* liver cell line, LV-1, used in this experiment was generated by the Berrimah Veterinary Laboratory (Department of Primary Industry and Resources, Government of Northern Territory), and was maintained at 28°C without CO_2_, in M199 media containing 10% FCS and antibiotics as outlined previously (11). The materials and methods used to stimulate LV-1 with poly dA:dT and low molecular weight (LMW) poly I:C (Invivogen, CA, USA) have been described previously (11). Three replicates per group were stimulated with poly dA:dT (dsDNA) and LMW poly I:C (dsRNA) at a concentration of 2 µg/mL, and cells were harvested at either 8, 24, 48, 72 or 96 h to examine the dynamic changes in host gene expression.

### RNA Extraction and RT-PCR

Total cellular RNA was isolated from each individual replicate and purified using an Isolate II RNA Mini Kit incorporating an on-column DNase treatment step (Bioline). The quality and quantity of the RNA was assessed using a NanoDrop spectrophotometer, and an Agilent 2100 Bioanalyzer (Agilent Technologies, USA). A260/280 ratios >1.8 and RNA integrity numbers >9.0 were standard for all total RNA samples purified across the stimulation time course. Initial synthesis of cDNA and subsequent real-time PCR (qPCR) were performed on an ABI 7000 as previously described using the primers and qPCR conditions, including the control genes GAPDH for the LV-1 cells, and primer sets for viperin, OASL, MX1, and ISG20L (11). Primers for CXCL10 were 5′-tgtgagcgccttgagatcat and 5′-gctgccacgtttagacttgtt; RTP2 5′-gtgacttcagcgagccagta and 5′-tccacggactctccatagca; ACOD1 5′-agtgggactactgggtagca and 5′-agaccatgcctagctgcatt.

### RNA-seq Library Construction and Sequencing

The protocol for RNA-seq library preparation was adapted from the Illumina TruSeq^®^ RNA sample preparation v2 Kit. Twelve strand-specific Illumina^®^ RNA-seq libraries were generated (three libraries, for each group of control; three replicates for dsRNA at the 24 h stimulation; and six replicates for each group of dsRNA and dsDNA at the 48 h stimulation) using 0.5 µg of total RNA. Total RNA was heated at 65°C for 5 min to denature any secondary structure and facilitate binding of the poly(A) RNA to the oligo-dT beads. Purification of poly(A) RNA was performed using Illumina TruSeq^®^ RNA sample preparation v2 Kit according to the manufacturer’s instructions (Illumina^®^ Inc., San Diego, CA, USA). Purified poly(A) RNA was then fragmented and primed using 19.5 µL of Elute, Prime and Fragment Mix containing random hexamers (Illumina^®^ Inc., San Diego, CA, USA) for 8 min at 94°C.

Synthesis of first-strand cDNA was performed immediately by incubating fragmented and primed mRNA with first strand master mix (Illumina, USA) and SuperScript^®^ II reverse transcriptase (Invitrogen™) at a ratio of 9:1. The reaction mixtures were run in a thermal cycler at 25°C for 10 min, at 42°C for 50 min, and 70°C for 15 min. Second-strand cDNA synthesis was initiated immediately, by adding 25 µL of second-strand master mix (Illumina^®^ Inc., San Diego, CA, USA) to each well, when the thermal cycle reached 4°C. After gentle and thorough mixing, the plate was incubated in a pre-heated thermal cycle at 16°C for 1 h. The double-stranded cDNA (ds cDNA) was subsequently purified by using AMPure XP beads (Invitrogen™, USA) according to the manufacturer’s instructions and eluted in 50 µL of the resuspension buffer (Illumina^®^ Inc., San Diego, CA, USA).

Blunt-end repair of ds cDNA was performed in a 100 µL reaction containing 10 µL diluted End Repair Control to 1/100 in resuspension buffer and 40 µL of End Repair Mix to each well (Illumina, USA). Reactions were incubated on a pre-heated thermal cycler at 30°C for 30 min and the ds cDNA was cleaned up using AMPure XP beads (Invitrogen™, USA) according to the protocol described in Illumina TruSeq^®^ RNA sample preparation v2 Kit, and eluted in 15 µL of the resuspension buffer (Illumina^®^ Inc., San Diego, CA, USA).

To facilitate Illumina^®^ adaptor ligation, a single “A” nucleotide was added to the 3′ ends of the blunt-end-repaired cDNA samples. Fifteen microliters of purified phosphorylated blunt-end-repaired cDNA was included in a final 30 µL reaction mixture containing 2.5 µL diluted A-tailing control to 1/100 in resuspension buffer and 12.5 µL of A-tailing mix to each well (Illumina^®^ Inc., San Diego, CA, USA). The reaction mixtures were run in a thermal cycler at 37°C for 30 min followed by 70°C for 5 min.

Illumina^®^ RNA-seq adaptor ligations were performed in a reaction volume of 37.5 µL containing 30 µL of phosphorylated blunt-ended cDNA in addition to 2.5 µL diluted ligation control, 2.5 µL ligation mix, and 2.5 of custom indexed adaptors to each well (see Table S1 in Supplementary Material for barcode index sequences). Reaction mixtures were incubated for 30°C for 10 min followed by inactivation of the ligation with 5 µL of stop ligation buffer into each well (Illumina^®^ Inc., San Diego, CA, USA). Adaptor-ligated cDNA was purified using AMPure XP beads (Invitrogen™, USA) according to the protocol described in Illumina TruSeq^®^ RNA sample preparation v2 Kit and eluted in resuspension buffer in a final volume of 20 µL.

PCR amplification of selectively enriched DNA fragments (50 µL) was performed using 20 µL of adaptor-ligated cDNA, 5 µL of Illumina^®^ PCR primer cocktail and 25 µL of Illumina^®^ PCR master mix to each well (Illumina^®^ Inc., San Diego, CA, USA). PCR amplification reactions were performed with the following temperature cycling profile: 98°C initial denaturation for 30 s; 15 cycles of 98°C for 10 s, 60°C for 30 s, and 72°C for 30 s; and 72°C final extension step for 5 min. PCR products were purified to remove PCR-generated adaptor-dimers using AMPure XP beads (Invitrogen™, USA) according to the protocol described in Illumina TruSeq^®^ RNA sample preparation v2 Kit with final elution in 30 µL of resuspension buffer.

All RNA-seq libraries were quantified and assessed using an Agilent Tape Station (Agilent Technologies) by the Australian Genomic Research Facility (AGRF, Melbourne) and confirmed insert sizes of 100–125 bp for all individual libraries. Individual RNA-seq libraries were standardized and pooled in equimolar quantities. The quantity and quality of the final pooled library was assessed as described above prior to sequencing by the facility. Cluster generation and sequencing of the pooled RNA-seq libraries were sequenced as paired-end using Illumina^®^ HiSeq HT chemistry according to the manufacturer’s instructions. RNA-seq data from this study have been deposited in the NCBI Sequence Read Achieve (SRA) under the accession number of PRJNA399550 (SUB2572918, SUB2982437) (http://www.ncbi.nlm.nih.gov/sra/).

### Transcriptome Data Analysis

An initial quality check was performed on each of the raw read files using FastQC (version 0.11.5) (23) to determine the best sequence read quality for filtering strategy. Low-quality reads were filtered considering the following criteria: (i) reads containing more than 25% bases with a phred score <20; (ii) reads with an average quality score <20; (iii) reads containing more than 10% of skipped bases (marked as “N”). The trimmomatic (version 0.32) (24) was used to remove the adapter sequences. The HiSat2 RNA-seq strand-specific aligner software package (version 2.0.5) with default parameters (25) was used to align filtered sequence reads to the most recent version of the *Crocodylus porosus* reference genome (assembly GCA_001723895.1 CroPor_comp1). Aligned sequence reads in individual BAM files were then used for a final quality check such as quality control of gene body coverage, duplication level, splice junction assessment, deletion, and mismatch profiles using RSeQC (version 2.6.4) (26), and all samples successfully passed.

The featureCounts tool, which is part of Subread software package (version 1.4.6p5) (27, 28), was used to quantify gene expression as reads count against *Crocodylus porosus* reference genome annotation (GCA_001723895.1 CroPor_comp1). Raw counts were loaded into the Bioconductor edgeR package (v 3.18.1) (29) and was used for the differentially expressed genes (DEGs) analysis. Expressed genes with a set threshold were defined as log_2_-count per million (logCPM) >0.5 in at least three different replicates. For each library, a trimmed mean of *M* values based normalization factor was calculated to eliminate composition biases between libraries (30). Furthermore, we also performed Voom transforms of RNA-seq data (31) for linear modeling, and it transformed count data to logCPM, which was used to estimate the mean variance relationships to compute the observation-level weights. The Bioconductor limma package was used to test for differential expression. Empirical Bayes method (ebayes) shrinkage was performed on the variances, and estimated moderated t-statistics and the associated *p* values. DEGs were defined by setting a false discovery rate (FDR) ≤0.01. Top 200 and 500 most variable genes were used to calculate a matrix of Euclidean distance for examining hierarchical clustering of samples in heat maps, using the heatmap.2 function of gplots (v 3.0.1).

### Functional Analysis of DEGs

Considering the lack of gene ontology (GO) for this non-model saltwater crocodile, we annotate GO term associated with crocodile protein coding genes by first comparing ortholog genes of *Gallus gallus* (chicken) as a reference organism and identified the molecular functions, biological processes, and cellular components for 8,399 genes, and then using human ortholog genes annotated another 2,928. TopGO (v 2.28.0) was used to perform the enrichment analysis on the DEGs with the total 11,327 genes as background. To characterize the identified genes from DEG analysis, a GO-based trend test was performed using Fisher’s exact test; *p* values <0.001 as a cut-off for statistically significant enrichment. To retrieve biological pathways related to the crocodile, we obtained KEGG orthologous gene information using the KEGG Automatic Annotation Server (32) with a eukaryote representative set as reference and default parameters, and DEG genes were linked to KEGG pathways using an in-house Perl script and edited based on KEGG Mapper results.

## Results

### Preliminary Analysis of Dynamic Changes in Selected Host Genes

**Figure 1:**
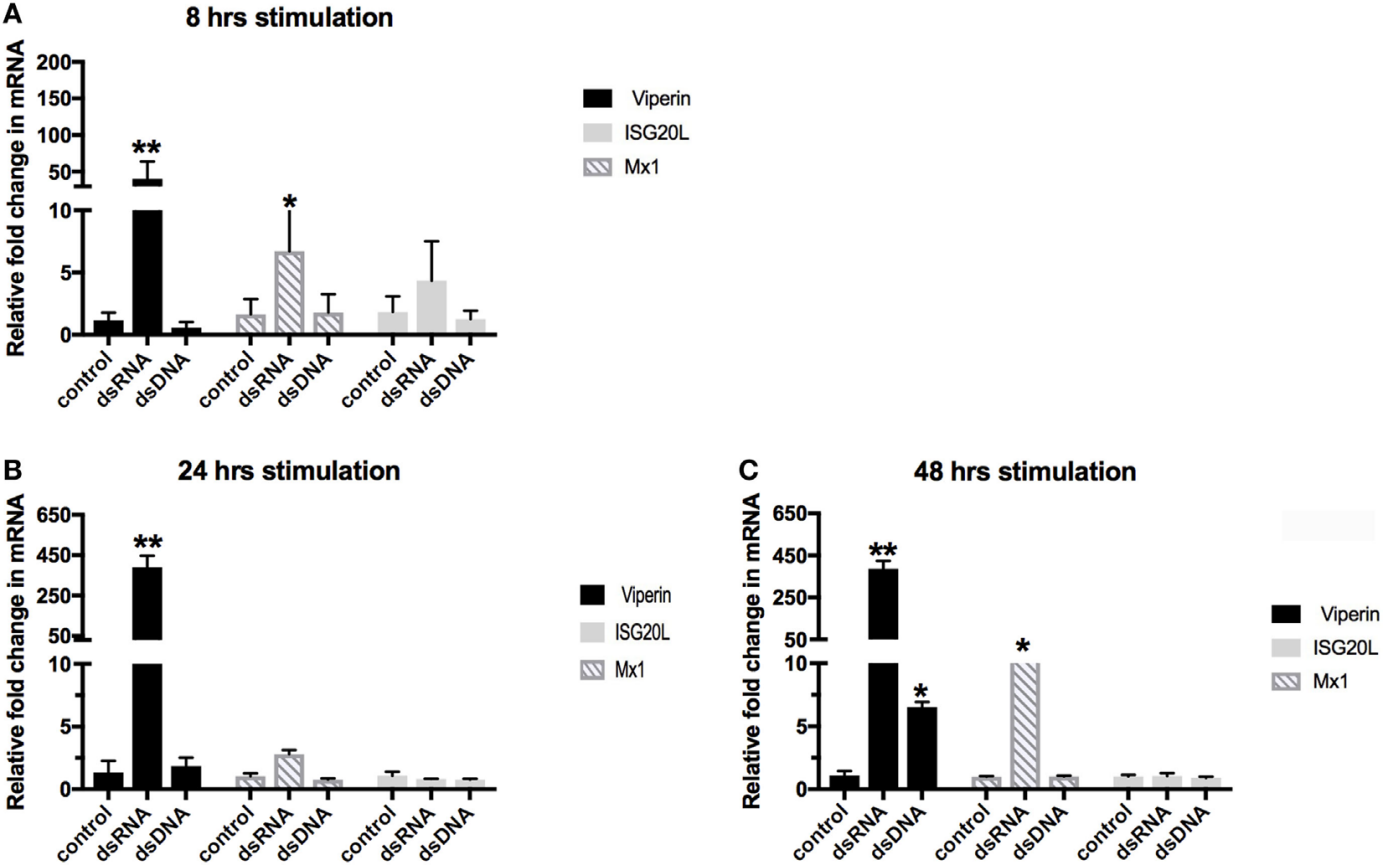
Time-course stimulation of LV-1 cells with dsRNA and dsDNA. Quantitative real-time PCR was performed to determine the changes in gene regulation of the ISGs. Relative expression levels of viperin, Mx1, and ISG20L were determined in comparison to GAPDH at 8 hrs **(A)**, 24 hrs **(B)** and 48 hrs **(C)**. The graph shows mean ± SD of values from three replicates per group. **Significant at the *p* < 0.001 level, *significant at the *p* < 0.05 level.

To better understand the ability of the crocodilian family members to elicit an early innate immune response to the recognition of dsRNA and dsDNA, we conducted a time-course stimulation of the *C*. *porosus* liver cell line, LV-1. LV-1 cells were stimulated for 8, 24, and 48 h to assess the ability of the *C*. *porosus* cells to activate PRRs, and initiate early innate signaling pathways to induce ISGs. A thorough examination of the *C. porosus* genome database for the presence of ISGs revealed assigned gene products with close homologies to other species of ISG20L, Mx1, and viperin. The selected genes were screened using primers and already established qPCR protocols on LV-1 cells (11), following 8, 24, and 48 h of stimulation with dsDNA and dsRNA. As can be seen in Figure 1, the ISG viperin responded strongest to both stimuli, with maximal expression following dsRNA plateauing at 24–48 h, and maximal expression following dsDNA stimulation seen at 48 h, albeit considerably lower than was observed for dsRNA stimulation (6-fold upregulation versus 387-fold upregulation, respectively). No observable response in ISG20L was seen for either stimuli, and a maximal increase in Mx1 mRNA to dsRNA was observed at 48 h (13-fold change) (Figure 1C). Stimulation of LV-1 cells with dsRNA induced the strongest response in ISG mRNA expression, which appeared to peak around 24–48 h, with a much weaker ISG response seen against dsDNA, which was maximal at 48 h post stimulation (hps).

### Preliminary Analysis of the High-Throughput RNA-seq Data

To further understand the dynamic gene expression profiles and their role in the immune pathway, RNA-seq was performed to explore the transcriptomes from crocodile LV-1 cells stimulated with the viral mimics, dsRNA and dsDNA at 24 and 48 hps, the times of maximal ISG expression in Figure 1. More than 63.21 million reads per library were generated, of which 58.87 million reads per library (93.21%) remained after adapter sequence and poor quality reads trimming (Table S1 in Supplementary Material). Alignment of the clean RNA-seq reads to the *C*. *porosus* reference genome yielded an average of 46.45 million reads (73.57%) per library uniquely mapped to the crocodile genome (Figure S1 in Supplementary Material). The RNA-seq read depth was distributed evenly along the whole body of the genes (Figures S2A,B in Supplementary Material), reflecting no obvious bias being introduced during randomly primed reverse transcription and subsequent RNA-seq.

### Analysis of Differential Gene Expression from RNA-seq Transcriptomes

**Figure 2:**
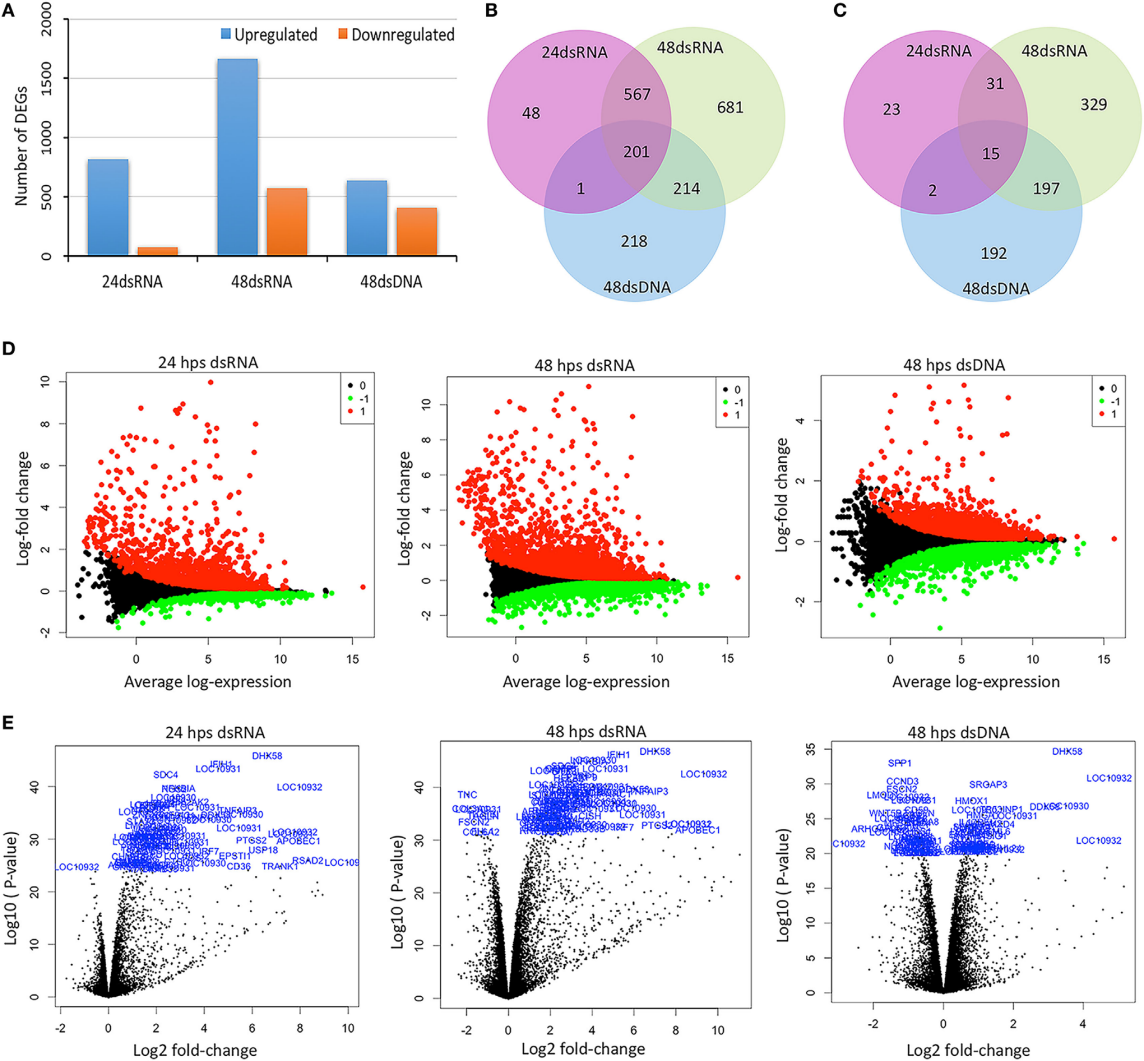
Overview of the RNA sequencing data under a time-course stimulation of LV-1 cells with dsRNA and dsDNA. LV-1 cells were harvested at 24 and 48 h post stimulation (hps). **(A)** Using a fold change threshold of ≥1.5 up or downregulated differentially expressed genes (DEGs) were identified from the comparison among control with dsRNA- and dsDNA-stimulated cells (DEGs were identified based on a false discovery rate *q*-value threshold of less than 0.05). **(B,C)** Venn diagrams showing of overlapping comparison of DEG profiles for dsRNA and dsDNA. The mRNA differential expressions in dsRNA- and dsDNA-stimulated LV-1 cells were depicted in three overlapping circles for 1.5-fold up **(B)** and down **(C)** regulation at 24 and 48 hps. **(D)** MA plots showing DEGs for dsRNA- and dsDNA-stimulated LV-1 cells. The *y*-axis represents the log fold change observed for each mRNA transcript and the *y*-axis represents the average-log expression values for each transcript. The upregulated genes are in red color, whereas downregulated genes are in green. Data for the genes that were not classified as differentially expressed are plotted as black. **(E)** Volcano plots showing DEGs for dsRNA- and dsDNA-stimulated LV-1 cells. The *x*-axis represents the log_2_ fold change observed for each mRNA transcript and the *y*-axis represents the log_10_ value of *p* values of the significant test between replicates for each transcript. Top 100 DEGs are highlighted in volcano plots.

Following the preliminary RNA-seq analysis, the sequence reads that mapped to unique locations in the *C. porosus* reference genome were used to quantify gene expression, compared between the control and the stimulated groups; and to list the DEGs with a FDR threshold of ≤0.05. Using a fold change threshold (±1.5), the total numbers of both up- and downregulated DEGs for dsRNA and dsDNA were calculated. A significantly greater number of upregulated genes than the number of downregulated genes was observed in both stimulated groups at all time points (Figure 2A). Comparison between the DEG profiles of dsRNA stimulated LV-1 cells revealed that 768 genes were co-upregulated in both the 24 and 48 h time points (Table S5 in Supplementary Material), indicating that the majority of genes induced by dsRNA viral mimics continue to increase in expression 24 hps (Figure 2B; Tables S5–S7 in Supplementary Material for gene lists corresponding to individual subgroupings within Figure 2B), with a total of 895 more DEGs being upregulated at 48 h compared to 24 hps (Table S11 in Supplementary Material for gene lists corresponding to individual subgroupings within Figure 2B). This pattern was also observed for downregulated genes following dsRNA viral mimic stimulation, with the highest number of genes being downregulated at the 48 h time point only (Figures 2C, 572 DEGs). Considerably lower numbers of genes (634 DEGs) were upregulated at 48 hps in dsDNA-stimulated LV-1 cells compared to stimulation with dsRNA at any time point; however, comparable levels of genes were downregulated at least 1.5-fold following stimulation with either dsDNA or dsRNA for 48 h (406 DEGs versus 572 DEGs, respectively, Figure 2C). Interestingly, 218 DEGs were uniquely upregulated and 192 DEGs uniquely downregulated in response to dsDNA viral mimics in the LV-1 cells, with 201 and 15 commonly DEGs up- or downregulated, respectively, between dsDNA and dsRNA viral mimics (Figures 2B,C, respectively) (all gene tables can be seen in Tables S2–S4 in Supplementary Material and Tables S12–S17 in Supplementary Material for gene lists corresponding to individual subgroupings within Figures 2B,C).

To illustrate the gene expression pattern across all individual biological samples at various time points within the stimulated groups, volcano plots (Figure 2D), and MA (mean average) plots (Figure 2E) were generated. There was a very good correlation between the fold change differences and *p*-value and/or average-log expression of the DEGs. In addition, DEGs were further visualized by a hierarchical clustering and an MDS (multi-dimensional scaling) plot was generated (Figures S3A,B in Supplementary Material). These data indicate a good clustering of samples according to the levels of similarities in the gene expression patterns, with a clear distinction between the dsRNA- and dsDNA-stimulated LV-1 cells, and importantly the gene expression profiles were able to differentiate between the two main groups of stimulated *C. porosus* LV-1 cells (Figure 3A in Supplementary Material). Furthermore, dsRNA- and dsDNA-stimulated samples at 24 and 48 hps were distinctly distributed and closely clustered according to the stimuli and time point (Figure 3B in Supplementary Material).

### Distinct and Dynamic Changes in Host DEGs in Response to dsRNA and dsDNA Stimulation

To further investigate the role of DEGs in host cells, we focused on the top-ranked 15 up- and downregulated genes from dsRNA- and dsDNA-stimulated cells (Tables 1–3). There was a significant increase in upregulation of immune-related genes in dsRNA-stimulated LV-1 cells at 48 h in comparison to 24 h (Table 2). Chemokine pathway-associated genes, such as C-X-C motif chemokine 10 (CXCL-10) and CXCL-11 were among the 15 most upregulated genes for dsRNA-stimulated LV-1 cells and were also top-ranked upregulated genes in dsDNA-stimulated cells at 48 hps (Table 3). Importantly, there was remarkable upregulation of innate immune regulatory and antimicrobial genes in both dsRNA- and dsDNA-stimulated LV-1 cells at 48 h, including the Radical S-adenosyl methionine domain-containing protein 2 (RSAD2), a well-documented broad antiviral gene, with immunomodulatory properties [reviewed in Helbig and Beard (33)], IFN-induced protein with tetratricopeptide repeats 5 (IFIT5) and probable ATP-dependent RNA helicase DDX60 (DDX60), both involved in the detection or enhancement of cytosolic RNA (34, 35) and aconitate decarboxylase 1 [ACOD1 or immune responsive gene 1], a relatively unexplored molecule that has been shown to have antimicrobial effects (36). Furthermore, programmed cell death 1 ligand 2 (PDCD1LG2) was found to be one of the most upregulated genes in the dsRNA- and dsDNA-stimulated LV-1 cells, although its expression was higher in dsRNA-stimulated cells than in dsDNA-stimulated cells (11.6 versus 6.58 log_2_ fold change); PDCD1LG2 is thought to limit viral-induced cellular damage due to overactive T-cells (37). Outside of the top 15 upregulated genes across data sets, many other molecules known to be pivotal to the early innate immune response to viral infection in mammals were commonly upregulated, as can be seen in the TLR and RIG-I pathways shown in Figures S4–S7 in Supplementary Material. Other important immune regulatory genes commonly upregulated were the interferon regulatory factors (IRF)1, 7, and 8; the type I interferon receptors IFNAR1/2; and the type I IFN, IFN-omega. The top 15 downregulated genes differed exclusively between dsRNA and dsDNA viral mimic simulation, with only one common DEG seen between both the stimulated LV-1 cells at the 24 and 48 h time points; a Rho-mediated GTP-binding protein Rho, a protein known to regulate intracellular actin dynamics. As might be expected, the up- and downregulation of gene transcripts stimulated at two different time points by dsRNA fell into three main categories, those that were differentially regulated initially presenting at 48 h only, and those that initially presented at 24 hps and then either continued to increase/decrease in expression, or remained unchanged in their expression between 24 and 48 h (genes demonstrating a significantly increased or decreased expression level between 24 and 48 h of dsRNA stimulation can be found in Tables S18 and S19 in Supplementary Material).

**Table 1:** The top 15 upregulated and downregulated genes for dsRNA stimulated versus control LV-1 cells at 24 h post stimulation as ranked by fold change and false discovery rate (FDR)-adjusted *P* values of ≤0.05.

Gene symbol	Gene name	log_2_FC	*P*-value	FDR-adjusted *P* values
LOC109323757	Programmed cell death 1 ligand 2	+10.52	1.40E−176	1.80E−174
LOC109309480	C-X-C motif chemokine 10	+9.62	0	0
–	Uncharacterized	+9.18	2.66E−68	1.68E−66
SELE	Selectin E	+8.81	0	0
ACOD1	Aconitate decarboxylase 1	+8.75	0	0
LOC109309482	C-X-C motif chemokine 11	+8.66	0	0
CD83	CD83 molecule	+8.46	0	0
LOC109309602	Probable ATP-dependent RNA helicase DDX60	+8.45	1.77E−43	7.51E−42
RSAD2	Radical S-adenosyl methionine domain containing 2	+8.32	0	0
LOC109320784	Cytosolic phospholipase A2 delta	+8.25	2.05E−37	7.51E−36
VCAM1	Vascular cell adhesion molecule 1	+7.96	1.27E−73	8.42E−72
LOC109308541	Receptor-transporting protein 2	+7.94	5.32E−92	4.40E−90
LOC109308610	C-C motif chemokine 20	+7.94	9.58E−32	3.05E−30
LOC109324007	Interferon-induced protein with tetratricopeptide repeats 5	+7.90	0	0
APOBEC1	Apolipoprotein B mRNA editing enzyme catalytic subunit	+7.89	0	0
POU2AF1	POU class 2 associating factor 1	−1.72	<0.05	<0.05
LOC109319006	Acid-sensing ion channel 2	−1.54	<0.05	<0.05
GFRA1	GDNF family receptor alpha 1	−1.51	3.53E−12	4.90E−11
DPP10	Dipeptidyl peptidase like 10	−1.37	<0.05	<0.05
LOC109323210	Inducible T-cell costimulator	−1.33	5.87E−134	6.26E−132
BRINP1	BMP/retinoic acid inducible neural specific 1	−1.26	9.31E−24	2.38E−22
RAB39A	RAS oncogene family	−1.25	<0.05	<0.05
KCNG3	Potassium voltage-gated channel modifier subfamily G member 3	−1.23	5.56E−26	1.50E−24
LOC109324336	Retinol dehydrogenase 16	−1.16	<0.05	<0.05
LOC109311611	Rho-related GTP-binding protein RhoB	−1.15	<0.05	<0.05
LGI3	Leucine rich repeat LGI family member 3	−1.11	<0.05	<0.05
GREM1	Gremlin 1	−1.09	5.86E−09	5.85E−08
C1QTNF7	C1q and tumor necrosis factor related protein	−1.07	8.87E−37	3.19E−35
LOC109324602	Mas-related G protein-coupled receptor member H	−1.06	<0.05	<0.05
LRRC17	Leucine rich repeat	−1.03	3.98E−08	3.55E−07

**Table 2:** The top 15 upregulated and downregulated genes for dsRNA stimulated versus control LV-1 cells at 48 h post stimulation as ranked by fold change and false discovery rate (FDR)-adjusted *P* values of ≤0.05.

Gene symbol	Gene name	log_2_FC	*P*-value	FDR-adjusted *P* values
LOC109323757	Programmed cell death 1 ligand 2	+11.60	0	0
LOC109320784	Cytosolic phospholipase A2 delta	+11.38	1.21E−215	8.14E−214
LOC109309480	C-X-C motif chemokine 10	+10.69	0	0
SELE	Selectin E	+10.51	0	0
CD83	CD83 molecule	+10.24	0	0
FLT3	Fms-related tyrosine kinase 3	+9.98	7.66E−113	2.64E−111
ACOD1	Aconitate decarboxylase 1	+9.82	0	0
RSAD2	Radical S-adenosyl methionine domain containing 2	+9.77	0	0
LOC109309506	Interleukin-8	+9.76	0	0
LOC109309482	C-X-C motif chemokine 11	+9.33	0	0
LOC109324007	Interferon-induced protein with tetratricopeptide repeats 5	+9.26	0	0
LOC109308809	Zinc finger protein RFP	+9.23	1.64E−60	2.98E−59
LOC109309602	Probable ATP-dependent RNA helicase DDX60	+9.05	6.45E−63	1.22E−61
LOC109308541	Receptor-transporting protein 2	+9.04	9.10E−181	5.28E−179
APOBEC1	Apolipoprotein B mRNA editing enzyme catalytic subunit 1	+9.01	0	0
PDLIM3	PDZ and LIM domain 3	−2.65	2.19E−24	1.70E−23
GPR20	G protein-coupled receptor 20	−2.50	7.39E−07	2.10E−06
CMKLR1	Chemerin chemokine-like receptor 1	−2.39	1.26E−63	2.43E−62
LOC109311611	Rho-related GTP-binding protein RhoB	−2.36	3.81E−06	1.01E−05
TLL1	Tolloid like 1	−2.14	2.06E−12	8.84E−12
LOC109320566	Potassium voltage-gated channel subfamily V member 2	−2.02	4.90E−12	2.04E−11
LOC109320408	Heat shock protein 30D	−1.99	8.49E−95	2.44E−93
WNT5B	Wnt family member 5B	−1.98	1.40E−214	9.37E−213
LMOD1	Leiomodin 1	−1.97	7.04E−264	5.52E−262
TNC	Tenascin C	−1.95	0	0
LOC109315030	Uncharacterized	−1.93	3.02E−142	1.39E−140
MUSTN1	Musculoskeletal 2C embryonic nuclear protein 1	−1.93	2.32E−05	5.68E−05
WISP1	WNT1 inducible signaling pathway protein 1	−1.93	3.00E−81	7.28E−80
FAM20A	FAM20A 2C Golgi-associated secretory pathway pseudokinase	−1.91	7.28E−35	7.86E−34
LOC109310616	Uncharacterized	−1.86	<0.05	<0.05

**Table 3:** The top 15 upregulated and downregulated genes for dsDNA-stimulated versus control LV-1 cells at 48 h post stimulation as ranked by fold change and false discovery rate (FDR)-adjusted *P* values of ≤0.05.

Gene symbol	Gene name	log_2_FC	*P*-value	FDR-adjusted *P* values
LOC109323757	Programmed cell death 1 ligand 2	+6.58	1.53E−13	1.36E−12
ACOD1	Aconitate decarboxylase 1	+5.22	5.13E−65	5.66E−63
LOC109308541	Receptor-transporting protein 2	+4.88	1.00E−11	7.44E−11
LOC109309480	C-X-C motif chemokine 10	+4.80	1.21E−196	1.08E−193
TRANK1	Tetratricopeptide repeat and ankyrin repeat	+4.72	7.90E−323	1.64E−319
LOC109324007	Interferon-induced protein with tetratricopeptide repeats 5	+4.68	0	0
RSAD2	Radical S-adenosyl methionine domain-2	+4.61	2.26E−122	7.84E−120
–	Uncharacterized	+4.54	<0.05	<0.05
LOC109309482	C-X-C motif chemokine 11	+4.51	2.54E−57	2.11E−55
LOC109321967	E3 ISG15-protein ligase HERC5	+4.44	0	0
CD83	CD83 molecule	+4.36	1.99E−42	9.43E−41
MOV10	Mov10 RISC complex RNA helicase	+3.89	6.86E−09	3.77E−08
SELE	Selectin E	+3.83	1.65E−35	5.63E−34
LOC109308809	Zinc finger protein RFP	+3.60	<0.05	<0.05
LOC109309602	Probable ATP-dependent RNA helicase DDX60	+3.60	<0.05	<0.05
LOC109320408	Heat shock protein 30D	−2.88	1.67E−158	1.10E−155
LOC109313754	Brain-specific homeobox/POU domain protein 3	−2.57	3.14E−09	1.80E−08
ACTC1	Actin	−2.12	<0.05	<0.05
BMP10	Bone morphogenetic protein 10	−2.00	<0.05	<0.05
CDH12	Adherin 12	−1.98	8.72E−17	9.91E−16
ARHGAP25	Rho GTPase activating protein	−1.90	1.37E−138	6.58E−136
MGAT5B	Mannosyl (alpha-1)-glycoprotein beta-1-*N*-acetyl-glucosaminyltransferase	−1.83	1.24E−15	1.29E−14
ARG1	Arginase-1	−1.81	9.77E−06	3.62E−05
LOC109319049	Golgin subfamily A member 6-like protein	−1.76	9.42E−09	5.07E−08
MORC1	MORC family CW-type zinc finger 1	−1.74	1.48E−10	9.83E−10
LMOD1	Leiomodin 1	−1.72	9.74E−220	1.10E−216
FAM198B	Family with sequence similarity 198 member B	−1.68	7.05E−35	2.33E−33
LOC109315946	Voltage-dependent T-type calcium channel subunit alpha-1I	−1.66	1.77E−06	7.28E−06
WNT5B	WNT family member 5B	−1.65	9.55E−163	7.01E−160
FOXO6	Forkhead box O6	−1.64	2.36E−33	7.44E−32

**Figure 3:**
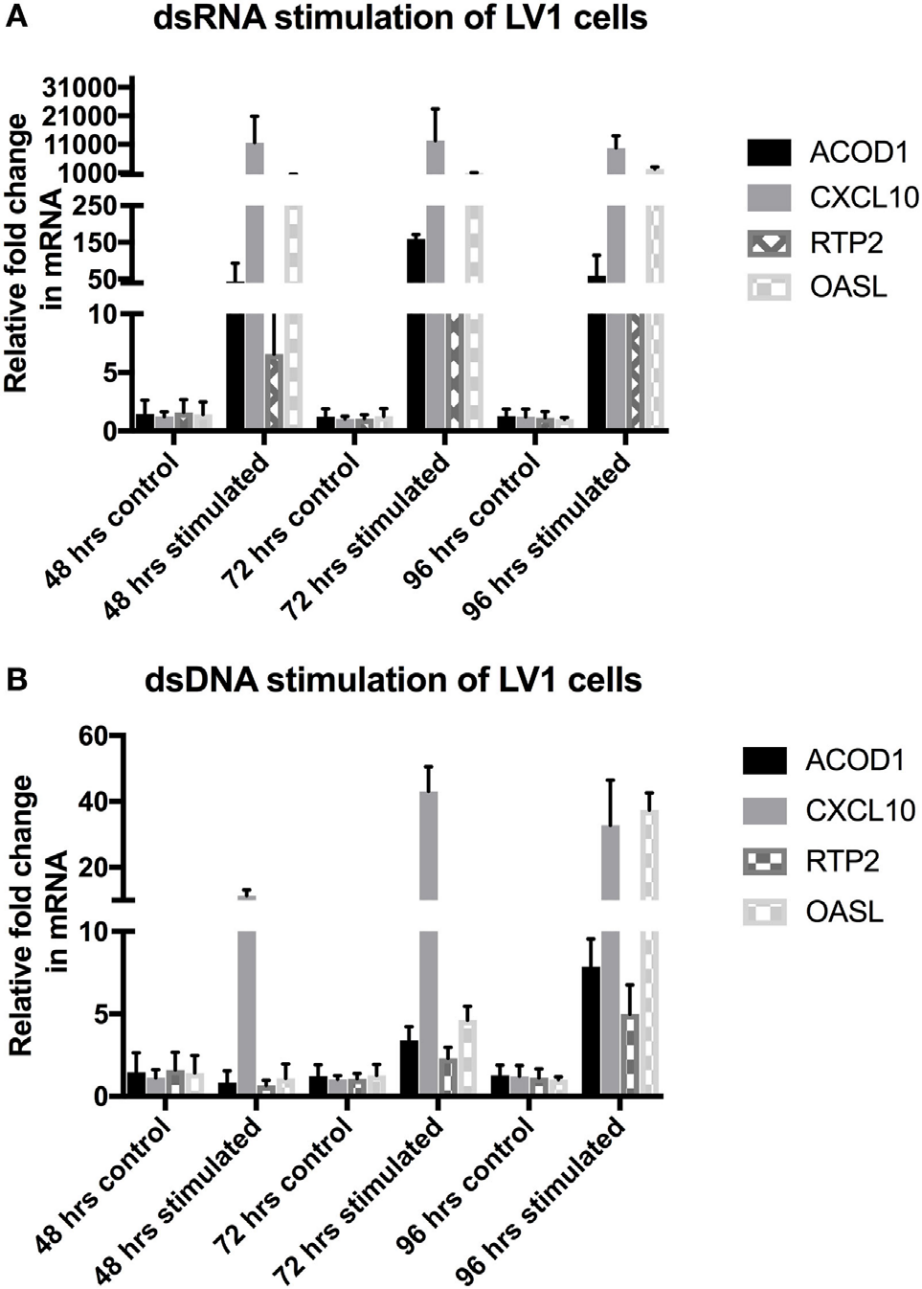
Post RNAseq validation with longer timecourse. Quantitative real-time PCR was performed to determine the changes in gene regulation of selected candidate genes. **(A,B)** Relative expression levels of aconitate decarboxylase 1 (ACOD1), CXCL10, RTP2, and OASL were determined following 48, 72, and 96 h of viral mimic stimulation. The graph shows mean ± SD of values from three replicates per group.

In order to validate the RNAseq data and to determine the longevity of the host innate response to viral mimic stimulation, we performed a longer time-course experiment, examining genes known to be involved in effective immune responses, and shown to be highly upregulated within the RNAseq data set for both dsRNA and dsDNA stimulation, including ACOD1, CXCL10, RTP2, and OASL. As can be seen in Figures 3A,B, both dsRNA and dsDNA viral mimic stimulation was able to significantly upregulate all selected candidate genes. Interestingly, dsRNA stimulation of LV-1 cells induced maximal expression of candidate genes between 48 and 72 h, with high expression still observed at 96 hps. In comparison, dsDNA stimulation induced a much slower response, with candidate genes showing maximal regulation at 96 h, excepting for CXCL10 (Figure 3B).

### Functional Categorization and Canonical Pathways of DEGs Detected with RNA-seq

**Figure 4:**
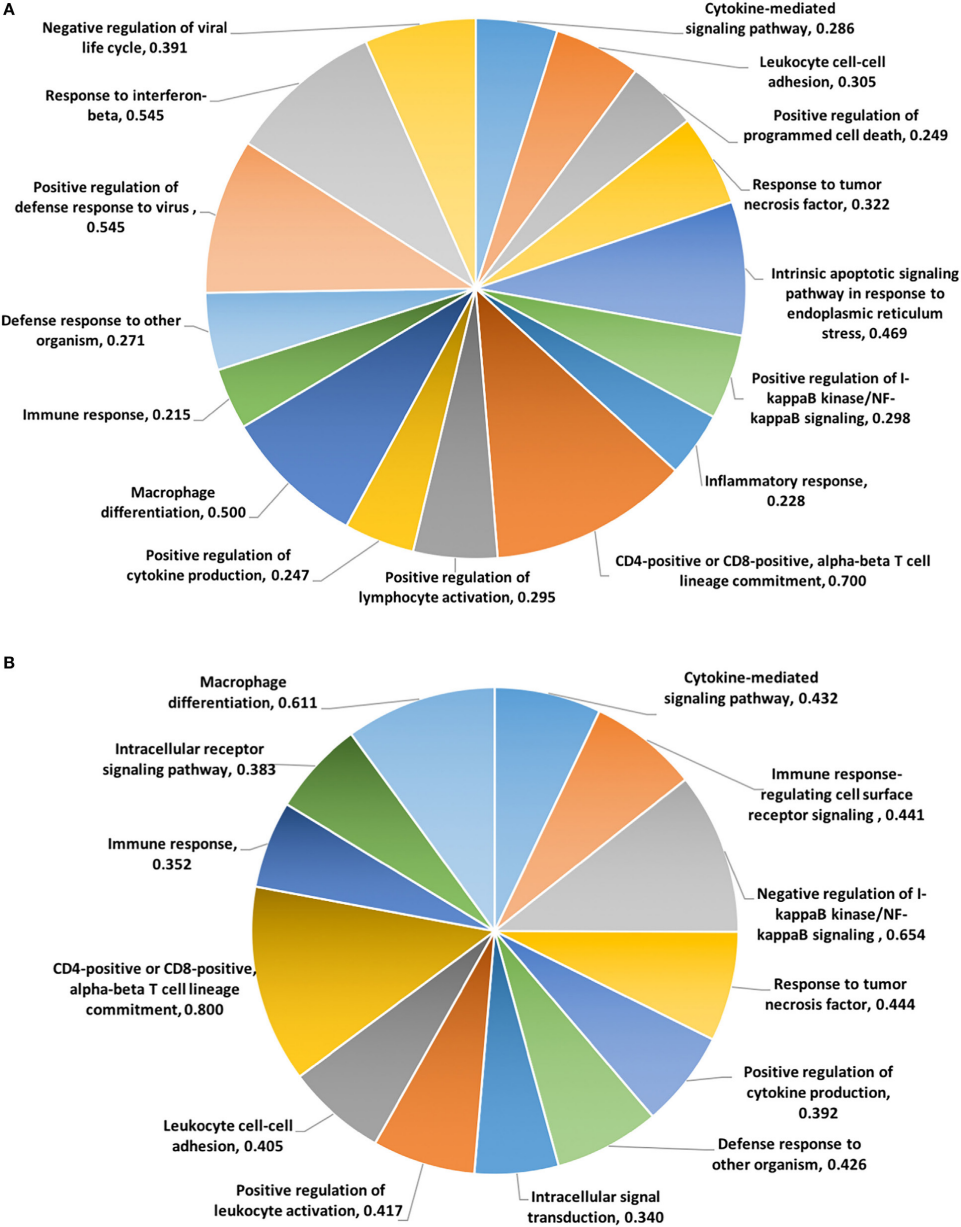
The top-ranked biological processes gene ontology functions identified by topGO package. Pie charts based on the DE genes involved in immunological function of the enriched biological processes generated using differentially expressed genes (DEGs) at 24 hps **(A)** and 48 hps **(B)**. The values for each function represent the ratio of DEGs versus the total annotated genes for each functional category.

To categorize functional networks and identify enriched biological process of GO terms, the DEGs at 24 and 48 hps were imported into the Bioconductor topGO package (38). There were 49 and 50 Biological Processes at 24 and 48 h activated significantly by dsRNA (Classic fisher’s test *P*-value ≤0.001) (Tables S20 and S21 in Supplementary Material). Figures 4A,B represents the list of activated Biological Processes involved in immunological function after LV-1 cells were stimulated with dsRNA at 24 and 48 hps, respectively. Among the top-ranked (Classic fisher’s test *P*-value ≤0.001) Biological Processes, *cytokine-mediated signaling pathway, CD4/CD8-positive alpha-beta lineage commitment, positive regulation of cytokine production, positive regulation of lymphocyte activation, immune response, and defense response to other organism* were activated both at 24 and 48 hps with dsRNA (Figures 4A,B). In addition, at 24 hps with dsRNA, there were other Biological Processes that were significantly over-represented including *I-kappaB kinase/NF-kappaB (IKK/NF-kB) signaling, inflammatory response, response to interferon-beta, negative regulation of viral life cycle, and positive regulation of defense response to virus* (Figure 4A). The significantly over-represented Biological Processes involved in immunological functions were relatively similar at both time points following dsRNA stimulation, excepting the presence of gene sets within the *negative regulation of I-kappaB kinase/NF-kappaB (IKK/NF-kB) signaling* at 48 hps with dsRNA (Figure 4B).

Analysis of the dsDNA viral mimic stimulation of LV-1 cells showed only 28 Biological Processes activated significantly (Classic fisher’s test *P*-value ≤0.001) at 48 hps with dsDNA (Table S22 in Supplementary Material). Among these, only three Biological Processes were related to immunological function, including, *cellular response to virus, defense response to virus*, and *negative regulation of viral process*, all known to have direct and/or indirect influence on the immune system.

**Figure 5:**
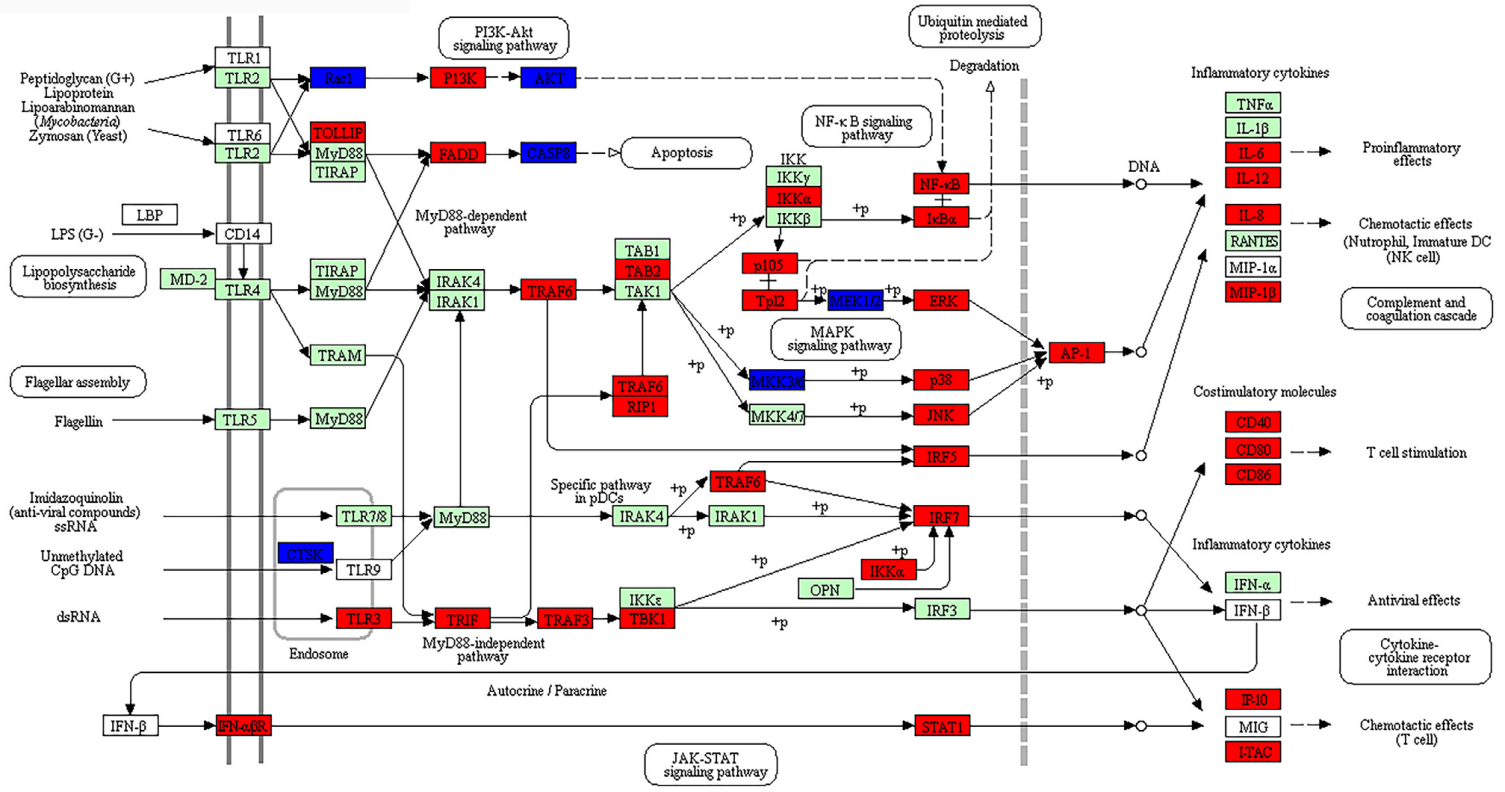
The enriched canonical pathway for toll-like receptor signaling at 48 hps with dsRNA. Pathway analysis using KEGG Mapper allowed us to identify the pathways that were differentially expressed between dsRNA-stimulated and non-stimulated LV-1 cells. Red and blue shading indicates increased and decreased expression, respectively, in dsRNA-stimulated LV-1 cells relative to the non-stimulated control cells. White and green shading indicates non-expression and non-differential expression, respectively. Solid and dashed lines represent direct and indirect interactions, respectively.

**Figure 6:**
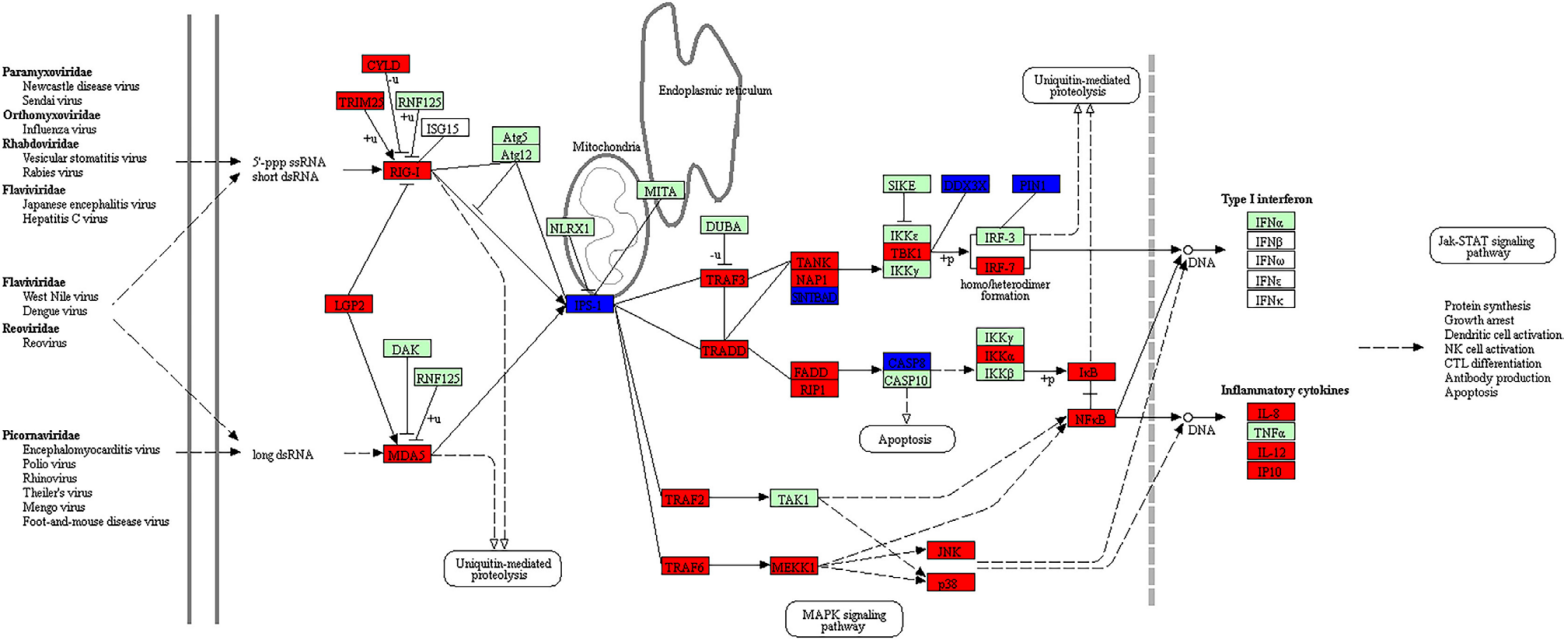
The enriched canonical pathway for RIG-I like receptor signaling at 48 hps with dsRNA. Pathway analysis using KEGG Mapper allowed us to identify the pathways that were differentially expressed between dsRNA-stimulated and non-stimulated LV-1 cells. Red and blue shading indicates increased and decreased expression, respectively, in dsRNA-stimulated LV-1 cells relative to the non-stimulated control cells. White and green shading indicates non-expression and non-differential expression, respectively. Solid and dashed lines represent direct and indirect interactions, respectively.

The enriched DEGs at both post-stimulated time points were also analyzed using KEGG Mapper (version 2.8) using the American Alligator (*Alligator mississippiensis;* amj) as a search model to identify canonical pathways. DEGs due to dsRNA and dsDNA stimulation were indicated as either red (upregulated) or blue (downregulated), with at least 30 hits required to consider the canonical pathways enriched. In this study, we identified 54 and 72 canonical pathways that were significantly enriched at 24 and 48 hps with dsRNA, respectively (Tables S23 and S24 in Supplementary Material), whereas 66 canonical pathways were identified at 48 hps with dsDNA (Table S25 in Supplementary Material). It is notable that a large number of DEGs were not found using the amj search model, although there were six separate canonical pathways identified at both time-course stimulations with dsRNA or dsDNA, which have immunological functions; five of these were common to all stimulation time points (highlighted as red in Tables S23–S25 in Supplementary Material). These canonical pathways included the *Toll-like receptor signaling pathway* which is shown to overlay with the DE gene expression results in Figure 5 (Figures S4 and S5 in Supplementary Material) and the *RIG-I-like receptor signaling pathway* which is presented in Figure 6 (Figures S6 and S7 in Supplementary Material). The notable differences included higher upregulation of key downstream signaling molecules such as activators of receptors (JAK-STAT signaling pathway) and activator of the TLR3 viral sensing (MyD88-independent pathway) in dsRNA- than dsDNA-stimulated LV-1 cells at 48 h; as well as the downstream signaling for the indirect P13K–Akt pathway being upregulated at 24 h following dsDNA stimulation (Figures S5 in Supplementary Material), whereas it was downregulated in the presence of dsRNA at 48 h (Figure 5).

We also further investigated the *RIG-I-like receptor signaling pathway* for dsRNA- and dsDNA-stimulated LV-1 cells at various time points. The RIG-I- and Mda5-mediated downstream signaling pathways, leading to the production of inflammatory cytokines and Type I IFN were activated to a greater extent in dsRNA stimulated LV-1 cells at 48 h (Figure 6) than at 24 h (Figures S6 in Supplementary Material), although the adaptor protein, IPS-1, which is known to be involved in RIG-I- and Mda5-mediated antiviral immune responses was downregulated at 48 hps.

## Discussion

In recent years, transcriptome profiling has enabled us to generate an unprecedented global view of the extent and complexity of gene transcription for a number of eukaryotic species, and has revealed a better understanding of the host genes and cellular pathways that are activated and perturbed in response to viral pathogens (39–43). Recent viral infections reported in the crocodile are novel, and do not have tissue culture models as yet. This study was aimed at describing the underlying host immune response against viral pathogens in this ancient animal using viral mimics, and the results have been demonstrated for the first time that stimulation of a primary passaged cell line from a crocodile (LV-1) is able to functionally upregulate multiple innate immune pathways, using RNA-seq analysis.

Innate immunity is a hosts first line of defense against viral infection, and is induced rapidly following detection of the pathogen. This response can include the production of antimicrobial peptides (44), complement, and lectins (45), however, the recognition of foreign viral products by PRRs, and the subsequent production of IFN remains the most potent host response to viral infection (46). Our knowledge of the signaling pathways following activation of the PRRs, as well as the downstream antiviral effector molecules, ISGs, induced by these pathways is based mostly on mammals, mainly humans and mice. Major explorations of antiviral innate immune responses in non-mammalian species remain very limited. There have been a number of studies in non-mammalian vertebrate species, such as the amphibians and the birds, describing the presence of various PRRs and IFNs in the genome, as well the potential role of varying IFN types; however, there is very little work analyzing whether these gene products are expressed in a functional context, or describing their antiviral effector genes for most species [reviewed in Chen et al. (47)]; with the exception of the chicken, which is an important agriculturally species (47, 48). The reptiles represent a bridge between the fish and mammals, and are the only ectothermic amniotes; this positions them as a pivotal species to not only enhance our evolutionary knowledge of the early innate systems controlling viral infection but also to provide insight into the complicated and intricate mammalian system that controls early viral infections.

The saltwater crocodile is the world’s largest living reptile species, and is evolved from the archosauria clade, which also includes the dinosaurs, pterosaurs, crocodilians, and birds; the latter two being the only living members (13). There a number of reported DNA and RNA viral infections of the crocodile, and this work describes the use of both DNA and RNA viral mimics at optimal time points, to stimulate the early innate responses in a recently derived crocodile cell line LV-1 (49). Many of the viruses infecting crocodiles are novel, and do not have tissue culture models as yet, likewise, the use of viral mimics ensured that we did not have to account for the ability of viruses to evade the innate immune system and disable varying arms of host protection (50). Using an RNA-seq approach and the recently annotated *C. porosus* genome as a reference, we were able to determine that the *C. porosus* host response to viral DNA and RNA mimics induced the expression of 634 and 1,663 genes respectively, with 406 and 572 genes being downregulated at 48 hps. There was considerable overlap between the two gene sets at 48 h, with 25 and 37% of genes up- or downregulated following dsRNA stimulation, being regulated similarly with dsDNA stimulation. Of these genes, approximately 10% belonged to uncharacterized open reading frames in the crocodile genome, currently unannotated, demonstrating the potential novelty of host proteins that may be involved in viral control in these animals.

RNA sequencing analysis was performed at two time points following RNA viral mimic stimulation, an early time point of 24 h, as well as the 48 h time point described above. These time points were chosen due to the early upregulation of initial ISGs tested prior to the RNA-seq experiment, in comparison to a distinct lack of ISG expression in the DNA viral mimic-stimulated cells at this point (Figure 1); in addition, ISGs have been demonstrated in mammalian cells previously to have distinct expression kinetics, with many genes being up- and downregulated within a 24 h period (51). In the context of the highest upregulated DEG’s, there was little difference between the 24 and 48 h stimulation in the LV-1 cells (Tables 1 and 2); however, there was only one common gene found to be downregulated in both time points following RNA viral mimic stimulation, Rho-related GTP-binding protein RhoB, a gene involved in intracellular protein trafficking, that has also previously been shown to be involved in enhanced entry of a select group of viruses (52).

The *C. porosus* genome annotation is an on-going project, as such much of the transcriptome remains unannotated, and currently there is no information regarding GO. Consequently, to understand the critical role of the DEGs in the biological processes, we annotated GO terms using ortholog genes of *Gallus gallus* (chicken) for 8,399 genes and human ortholog genes for another 2,928. As expected, GO-term analysis revealed a marked increase in biological processes associated with immunological cell functions in all groups stimulated with nucleic acid viral mimics, including “*cellular response to virus*,” “*defence response to virus*,” and “*immune response*,” with general immunological processes being over-represented in all sample sets (Tables S20–22 in Supplementary Material). Interestingly, the biological processes involved in the set of DEGs from the DNA viral mimic stimulated cells demonstrated significantly less pathways common to immune processes in comparison to the RNA viral mimic-stimulated cells. However, in terms of pathway analysis, utilizing KEGG mapper, the canonical pathways found to be significantly represented in both DNA and RNA viral mimic-stimulated cells were remarkably similar (Tables S23–25 in Supplementary Material).

Many common innate immune pathways known to be involved in the recognition of both viral dsDNA and dsRNA in mammals in the cytosol were all shown to be significantly represented in the crocodile cell lines stimulated with the viral replication mimics. Canonical pathway analysis demonstrated the MAPK signaling pathway to be significantly represented in all three datasets, being in the top five canonical pathways (Tables S23–25 in Supplementary Material). This signaling pathway has previously been shown to be pivotal in the upregulation of inflammatory genes following activation of both dsRNA and dsDNA signaling pathways after host viral recognition (53, 54). RNA viral mimic stimulation was shown to activate gene sets present in both the RIG-I and TLR3 signaling pathways, the predominant pathways known to sense viral RNA (54). All pathway members required to induce IFN production were positively regulated, with the exception of IRF3 and IPS-1, which was downregulated at the 48 h RNA time point. Both of these proteins are known to be present at high basal levels, and are activated *via* phosphorylation alone, or phosphorylation and ubiquitination, respectively, following viral sensing by RIG and TLR3 (55–57). Stimulation of LV-1 cells with the DNA viral mimics also induced gene sets common to the RIG-I and TLR signaling pathways, although as many of these genes are inducible *via* type I IFN production (discussed below), a likely result of DNA stimulation, it is not surprising that they are differentially regulated. Although essentially an RNA sensor of 5′ tri-phosphate ssRNA, RIG-I has previously been reported to detect viral DNA following modifications *via* RNA polymerase III (58) in human and murine cell lines, and it is possible that non-mammalian host cells may also perform this function, but further work will need to be done to confirm this.

No prior work has been performed to identify functional dsDNA receptors in non-mammalian vertebrates, however the *C. porosus* genome contains both cGAS and DDX41, two known mammalian receptors for dsDNA sensing in the cytosol [reviewed in Paludan and Bowie (59)], as well as TLR21, which is known to act as a functional homolog of TLR9 in the chicken (60). Interestingly, the crocodile genome appears to lack an open reading frame for STING (or MITA), the signaling adaptor that has been shown to be central to the upregulation of the antiviral cytokines, type I and III IFN, following viral DNA sensing by all dsDNA receptors, even though it is present in the very closely related genomes of the American alligator and the gharial [reviewed in Burdette and Vance (61)]. However, presumably TLR21 signals through a similar pathway to the other endosomal TLRs, which appear to be represented in the significant canonical pathways following both RNA and DNA viral mimic stimulation (Tables S23–25 in Supplementary Material). Further detailed studies will be required to elucidate the functional source of ISG induction following DNA stimulation in the crocodile, and the specific pathways involved.

The activation of PRR pathways *via* the sensing of viral nucleic acid, results in the production of the antiviral cytokines, termed IFNs. It is these IFNs that are known to induce the upregulation of hundreds of ISGs *via* the JAK–STAT pathway, in response to viral infection, many of which are known to be potently antiviral in the context of mammals (3, 4). The *C. porosus* genome contains one documented open reading frame with a similarity to the type I IFN-omega. This gene product was significantly upregulated during stimulation with RNA viral mimics at both the early and late time points (Tables S2 and S3 in Supplementary Material), and is a probable candidate for activation of the JAK/STAT pathway in the crocodile, *via* the type I INFA receptors. There were also multiple highly upregulated known antiviral ISGs present in both RNA and DNA viral mimic stimulated LV-1 cells, including viperin (RSAD-2), MX1, MOV10, CH25H, ADAR, DDX58/60, IRF1/7, ISG15 and TRIM5/25 [reviewed in Schoggins and Rice (4)].

This work is demonstrated for the first time that stimulation of a primary passaged cell line from a crocodile, or perhaps any reptile, is able to functionally upregulate multiple PPR pathway members, and, subsequently, induce a large sub-set of genes with potential antiviral function in response to viral mimics. We have also shown that multiple conserved canonical pathways are likely in play in the host response to viral infection in this reptile, with many of the viral-responsive genes being novel (approximately 10%). Further work needs to be performed to determine the functionality of potential important players in the antiviral innate host response of the reptile to a viral infection. This work will underpin these studies, and offers a better understanding of these pathways and the effector genes responsible for control of viral infection in non-mammalian species. An enhanced knowledge of these ancient antiviral pathways will not only add to our understanding of the host antiviral innate response in non-mammalian species, but is critical to fully comprehend the complexity of the mammalian innate immune response to viral infection.

## Author Contributions

SS and KH conceived and designed the experiments. SS performed the experiments. BW-S performed the post validation of RNAseq data using some selected candidate genes. SS and YW analyzed the data. SS and KH contributed reagents and materials. SS and KH wrote the paper. All authors read and approved the final manuscript.

## Conflict of Interest Statement

The authors declare that the research was conducted in the absence of any commercial or financial relationships that could be construed as a potential conflict of interest.
