# Supplementary material for: Dynamic Changes in Host Gene Expression following In Vitro Viral Mimic Stimulation in Crocodile Cells
Source: Front Immunol. 2017 Nov 22;8:1634. doi: 10.3389/fimmu.2017.01634 (PMC5702629; doi:10.3389/fimmu.2017.01634)
Supplement: Supplementary file 1 [file Data_Sheet_1.zip › Data sheet 1/Supplementary Material.pdf]

Supplementary Material

Dynamic Changes in Host Gene Expression Following *in-Vitro* Viral Mimic Stimulation in Crocodile Cells

Subir Sarker[1], Yinan Wang[2], Brenden Warren-Smith[1], Karla J Helbig[1]*

[1] Department of Physiology, Anatomy and Microbiology, School of Life Sciences, La Trobe University, Melbourne, VIC 3086, Australia
[2] Genomic Research Platform, La Trobe University, Melbourne, VIC 3086, Australia

*Correspondence: Dr Karla Helbig; k.helbig@latrobe.edu.au

Supplementary Figures

Supplementary Figure S1

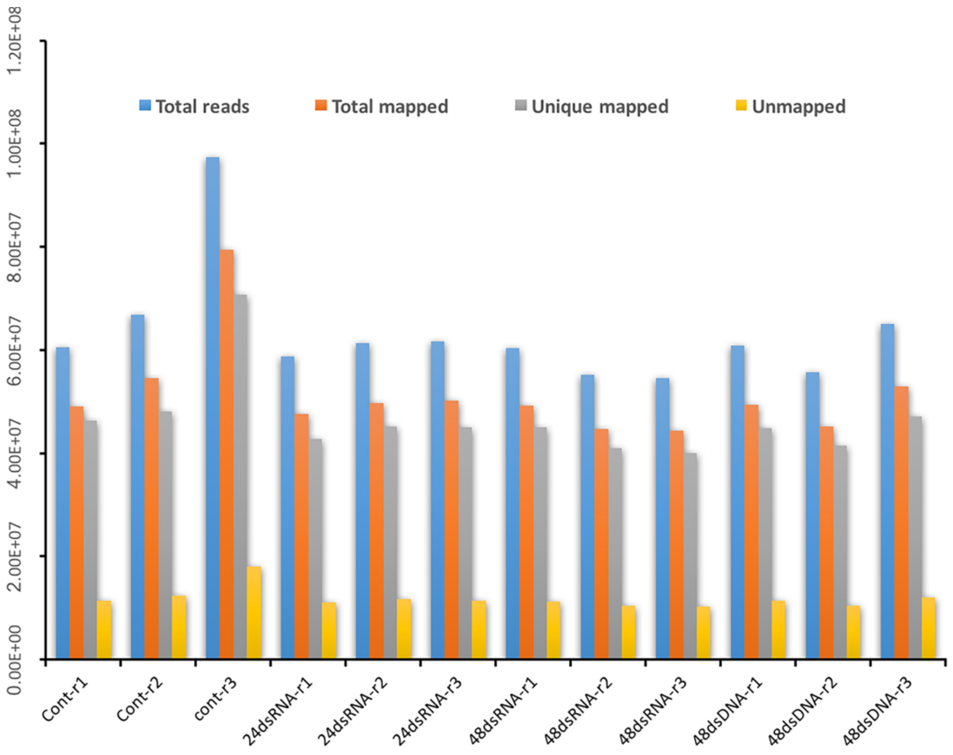

Supplementary Figure S1: Overall mapping Summary. Total reads and mapping status for all samples. Detailed statistics are presented in Table S1.

Supplementary Figure S2

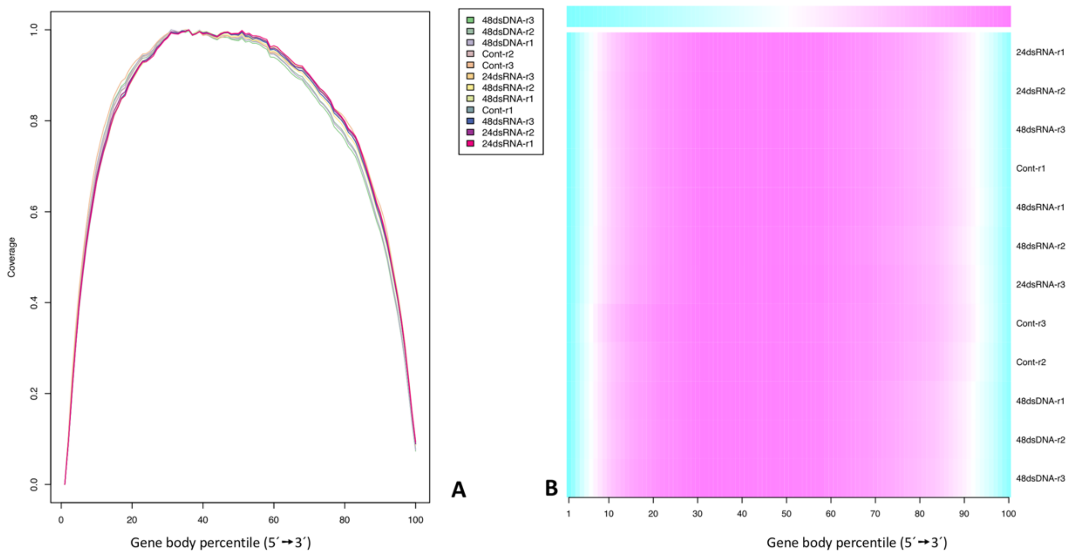

Supplementary Figure S2: Calculation of the RNA-seq reads coverage over gene body. LineGraphs were generated for all BAM files (A), with heat maps generated where three or more BAM files were provided (B). The RNA-seq read depth was distributed evenly along the whole body of the genes

Supplementary Figure S3

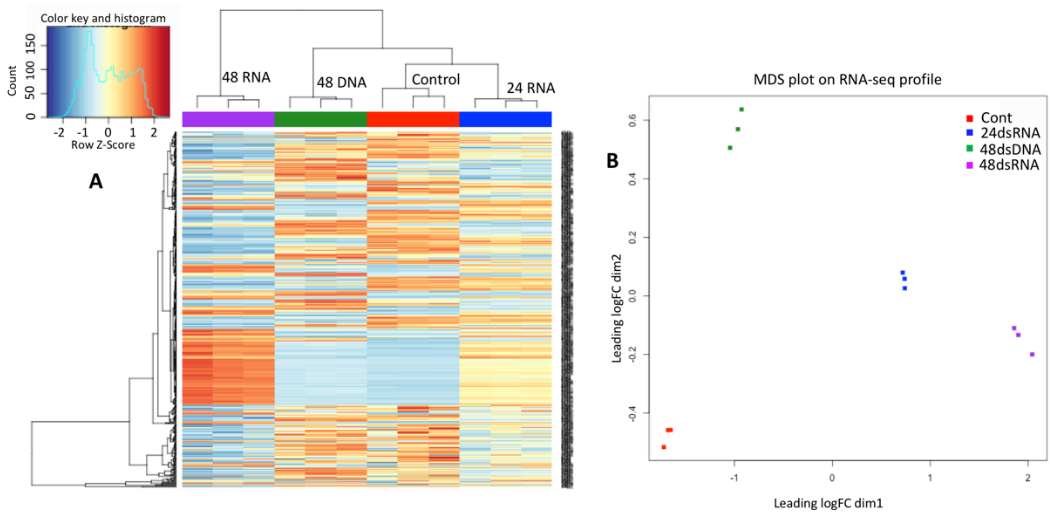

Supplementary Figure S3: Examination of gene expression pattern across all individual biological samples at various time-points. Hierarchical clustering was done by calculating a matrix of Euclidean distances from log(FPKM) for the 500 most variable genes. Hierarchical clustering (A) and multi-dimensional scaling (MDS) plots (B) indicate a good relationship of samples according to the levels of similarities in the gene expression patterns.

Supplementary Figure S4

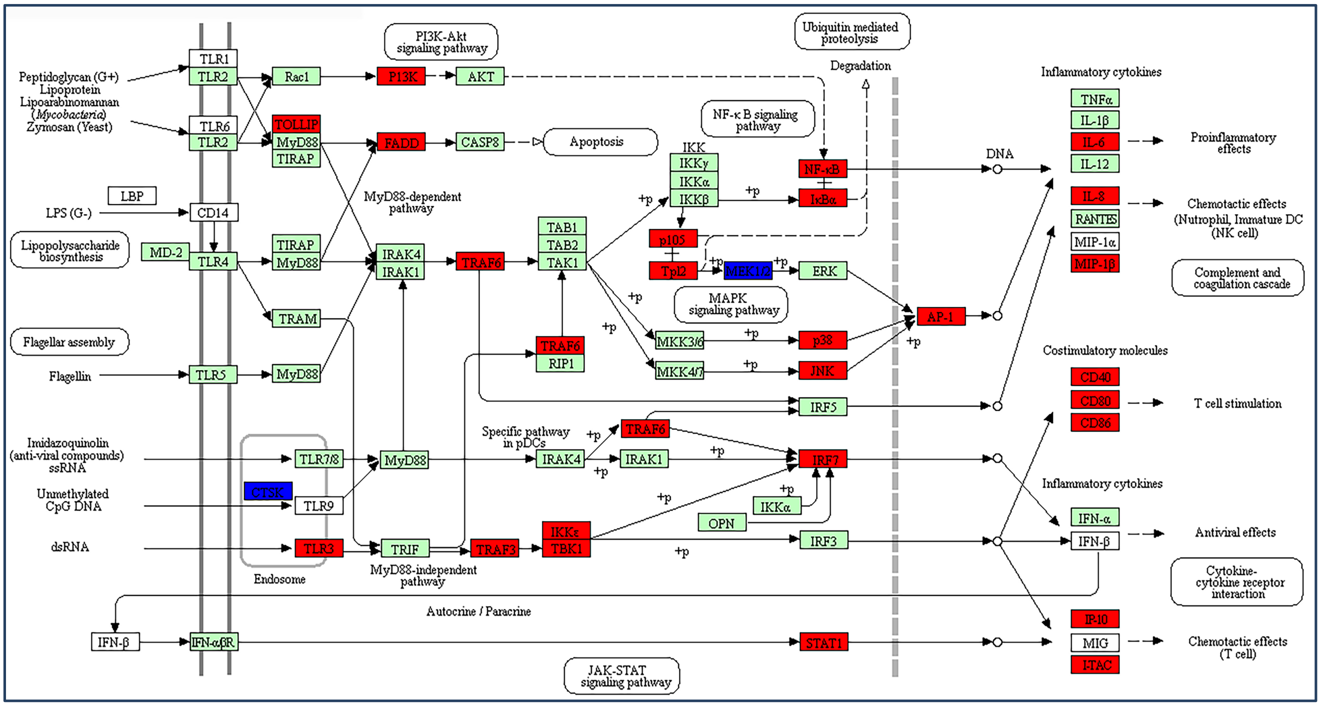

Supplementary Figure S4: The enriched canonical pathway for Toll-like receptor signalling at 24 hps with dsRNA. Pathway analysis using KEGG Mapper allowed us to identify the pathways that were differentially expressed between dsRNA stimulated and non-stimulated LV-1 cells. Red and blue shading indicates increased and decreased expression, respectively in dsRNA stimulated LV-1 cells relative to the non-stimulated control cells. White and green shading indicates non-expression and non-differential expression, respectively. Solid and dashed lines represent direct and indirect interactions, respectively.

Supplementary Figure S5

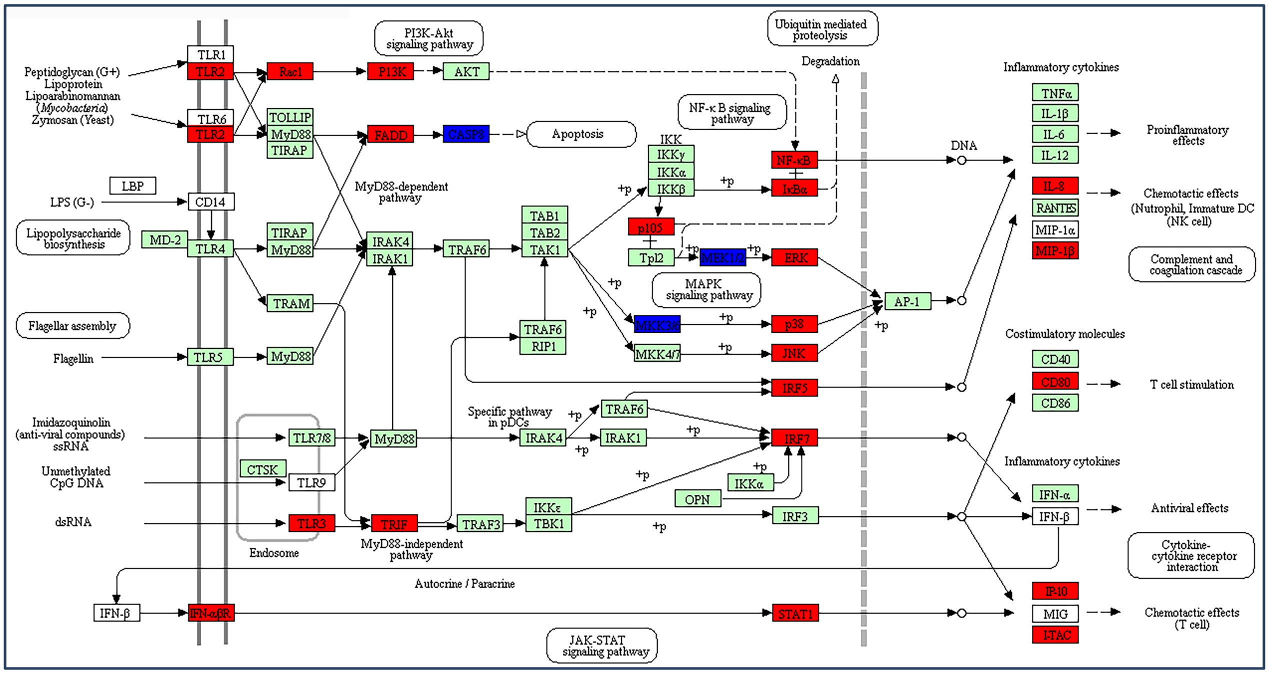

Supplementary Figure S5. The enriched canonical pathway for Toll-like receptor signalling at 48 hps with dsDNA. Pathway analysis using KEGG Mapper allowed us to identify the pathways that were differentially expressed between dsRNA stimulated and non-stimulated LV-1 cells. Red and blue shading indicates increased and decreased expression, respectively in dsRNA stimulated LV-1 cells relative to the non-stimulated control cells. White and green shading indicates non-expression and non-differential expression, respectively. Solid and dashed lines represent direct and indirect interactions, respectively.

Supplementary Figure S6

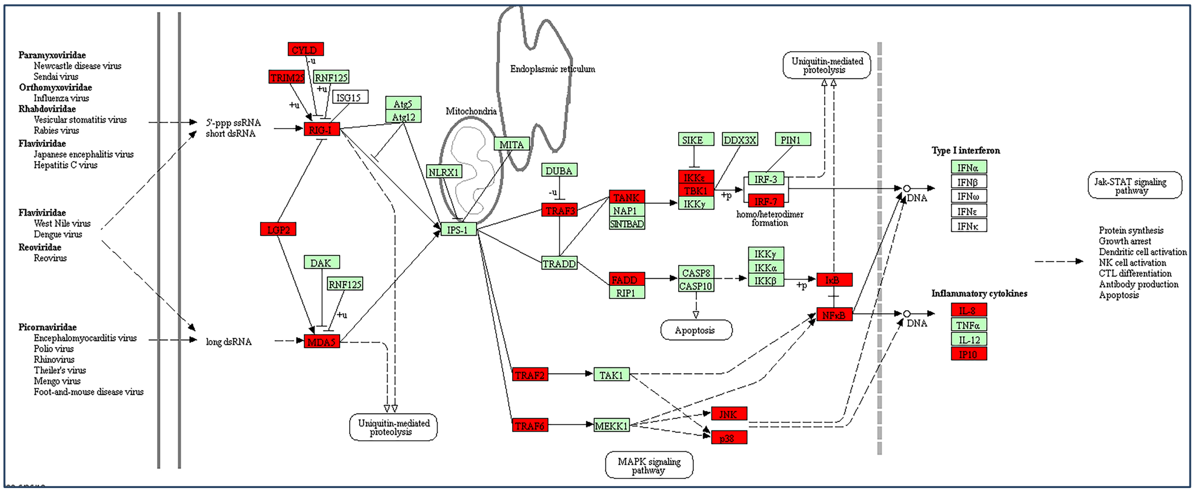

Supplementary Figure S6: The enriched canonical pathway for RIG-I like receptor signalling at 24 hps with dsRNA. Pathway analysis using KEGG Mapper allowed us to identify the pathways that were differentially expressed between dsRNA stimulated and non-stimulated LV-1 cells. Red and blue shading indicates increased and decreased expression, respectively in dsRNA stimulated LV-1 cells relative to the non-stimulated control cells. White and green shading indicates non-expression and non-differential expression, respectively. Solid and dashed lines represent direct and indirect interactions, respectively.

Supplementary Figure S7

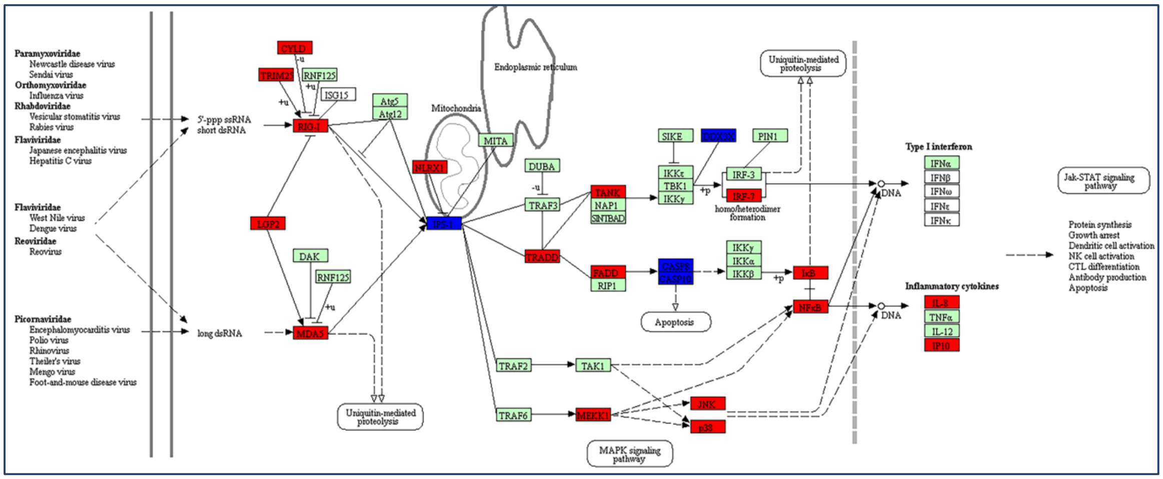

Supplementary Figure S7: The enriched canonical pathway for RIG-I like receptor signalling at 48 hps with dsDNA. Pathway analysis using KEGG Mapper allowed us to identify the pathways that were differentially expressed between dsRNA stimulated and non-stimulated LV-1 cells. Red and blue shading indicates increased and decreased expression, respectively in dsRNA stimulated LV-1 cells relative to the non-stimulated control cells. White and green shading indicates non-expression and non-differential expression, respectively. Solid and dashed lines represent direct and indirect interactions, respectively.

Supplementary Tables

Table S1: RNA-seq library information (alignment and assignment of reads) for dsRNA and dsDNA stimulated LV1 crocodile cells. **[Table_S1.xlsx]**

Table S2: Detailed information for DE genes detected using RNA-seq in dsRNA stimulated LV-1 cells versus control samples at 24 hps; using a fold change threshold of >1.5, P-value of ≤ 0.05 and FDR-adjusted P values of ≤ 0.05. **[Table_S2.xlsx]**

Table S3: Detailed information for DE genes detected using RNA-seq in dsRNA stimulated LV-1 cells versus control samples at 48 hps; using a fold change threshold of >1.5, P-value of ≤ 0.05 and FDR-adjusted P values of ≤ 0.05. **[Table_S3.xlsx]**

Table S4: Detailed information for DE genes detected using RNA-seq in dsDNA stimulated LV-1 cells versus control samples at 48 hps; using a fold change threshold of >1.5, P-value of ≤ 0.05 and FDR-adjusted P values of ≤ 0.05. **[Table_S4.xlsx]**

Table S5: Detailed information for commonly upregulated genes detected in dsRNA stimulated LV-1 cells between 24 and 48 hps; using a fold change threshold of >1.5, P-value of ≤ 0.05 and FDR-adjusted P values of ≤ 0.05. **[Table_S5.xlsx]**

Table S6: Detailed information for commonly upregulated genes detected betwwen dsRNA and dsDNA stimulated LV-1 cells at 24 and 48 hps, respectively; using a fold change threshold of >1.5, P-value of ≤ 0.05 and FDR-adjusted P values of ≤ 0.05. **[Table_S6.xlsx]**

Table S7: Detailed information for commonly upregulated genes detected betwwen dsRNA and dsDNA stimulated LV-1 cells at 48 hps; using a fold change threshold of >1.5, P-value of ≤ 0.05 and FDR-adjusted P values of ≤ 0.05. **[Table_S7.xlsx]**

Table S8: Detailed information for commonly downregulated genes detected in dsRNA stimulated LV-1 cells between 24 and 48 hps; using a fold change threshold of >1.5, P-value of ≤ 0.05 and FDR-adjusted P values of ≤ 0.05. **[Table_S8.xlsx]**

Table S9: Detailed information for commonly downregulated genes detected betwwen dsRNA and dsDNA stimulated LV-1 cells at 24 and 48 hps, respectively; using a fold change threshold of >1.5, P-value of ≤ 0.05 and FDR-adjusted P values of ≤ 0.05. **[Table_S9.xlsx]**

Table S10: Detailed information for commonly downregulated genes detected betwwen dsRNA and dsDNA stimulated LV-1 cells at 48 hps; using a fold change threshold of >1.5, P-value of ≤ 0.05 and FDR-adjusted P values of ≤ 0.05. **[Table_S10.xlsx]**

Table S11: Detailed information for total upregulated genes detected in dsRNA stimulated LV-1 cells at 48 hps; using a fold change threshold of >1.5, P-value of ≤ 0.05 and FDR-adjusted P values of ≤ 0.05. **[Table_S11.xlsx]**

Table S12: Detailed information for uniquely upregulated genes detected in dsRNA stimulated LV-1 cells at 24 hps; using a fold change threshold of >1.5, P-value of ≤ 0.05 and FDR-adjusted P values of ≤ 0.05. **[Table_S12.xlsx]**

Table S13: Detailed information for uniquely upregulated genes detected in dsRNA stimulated LV-1 cells at 48 hps; using a fold change threshold of >1.5, P-value of ≤ 0.05 and FDR-adjusted P values of ≤ 0.05. **[Table_S13.xlsx]**

Table S14: Detailed information for uniquely upregulated genes detected in dsDNA stimulated LV-1 cells at 48 hps; using a fold change threshold of >1.5, P-value of ≤ 0.05 and FDR-adjusted P values of ^{2} 0.05. **[Table_S14.xlsx]**

Table S15: Detailed information for uniquely downregulated genes detected in dsRNA stimulated LV-1 cells at 24 hps; using a fold change threshold of >1.5, P-value of ≤ 0.05 and FDR-adjusted P values of ≤ 0.05. **[Table_S15.xlsx]**

Table S16: Detailed information for uniquely downregulated genes detected in dsRNA stimulated LV-1 cells at 48 hps; using a fold change threshold of >1.5, P-value of ≤ 0.05 and FDR-adjusted P values of ≤ 0.05. **[Table_S16.xlsx]**

Table S17: Detailed information for uniquely downregulated genes detected in dsDNA stimulated LV-1 cells at 48 hps; using a fold change threshold of >1.5, P-value of ≤ 0.05 and FDR-adjusted P values of ≤ 0.05. **[Table_S17.xlsx]**

Table S18: Gene transcripts significantly upregulated post dsRNA stimulation between 24 and 48 hours. **[Table_S18.xlsx]**

Table S19: Gene transcripts significantly downregulated post dsRNA stimulation between 24 and 48 hours. **[Table_S19.xlsx]**

Table S20: Biological Processes identified using topGO for DE genes detected using RNA-seq from dsRNA-stimulated versus non-stimulated control LV-1 cells at 24 hps. **[Table_S20.xlsx]**

Table S21: Biological Processes identified using topGO for DE genes detected using RNA-seq from dsRNA-stimulated versus non-stimulated control LV-1 cells at 48 hps. **[Table_S21.xlsx]**

Table S22: Biological Processes identified using topGO for DE genes detected using RNA-seq from dsDNA-stimulated versus non-stimulated control LV-1 cells at 48 hps. **[Table_S22.xlsx]**

Table S23: Canonical pathways identified using KEGG Mapper for DE genes detected using RNA-seq from dsRNA-stimulated versus non-stimulated control LV-1 cells at 24 hps (sorted by the number of hits). **[Table_S23.xlsx]**

Table S24: Canonical pathways identified using KEGG Mapper for DE genes detected using RNA-seq from dsRNA-stimulated versus non-stimulated control LV-1 cells at 48 hps (sorted by the number of hits). **[Table_S24.xlsx]**

Table S25: Canonical pathways identified using KEGG Mapper for DE genes detected using RNA-seq from dsDNA-stimulated versus non-stimulated control LV-1 cells at 48 hps (sorted by the number of hits). **[Table_S25.xlsx]**
